# Unfair Discrimination

**Published:** 1912-04

**Authors:** S. Baruch

**Affiliations:** 51 W. 70th St.


### Unfair Discrimination.

51 W. 70th St., March 25, 1912.

Dear Dr. Benedict:

I hope the inclosed may move you to advocate concerted action by medical societies to obtain a decision by the highest courts on this flagrant injustice of state laws against educated practitioners.

Yours sincerely,
S. Baruch.

---

Dr. Baruch's inclosure consists of a very friendly editorial in the *N. Y. Sun* of March 17, 1912, entitled The Physician and the Public, and deprecating the unfairness of the present laws in many states in which the rigid requirements for the regular physician are placed side by side with easy conditions for all sorts of charlatans. "It is indisputable that the physician is the only real altruist in any community" says the *Sun* (of course, only in the sense of a type of a profession). Reference is also made to many acts of medical heroism and to the customary charity of the profession.

We are strongly of the opinion that a fair deal, all over the country, for every man, will not be obtained till we have one set of laws as well as one flag. The license laws, for example, are most unjust and can scarcely be placed on a fair basis by state legislation.

There is one thought that occurs to us regarding the particular matter to which Dr. Baruch alludes: We do not want to lower the requirements for the regular medical student, graduate and licenciate. If it is necessary, in response to the specious plea of fairness and broad-mindedness, to provide for the license of Christian Scientists, osteopaths, optometrists and "that all tribe" as it is so well expressed in Latin, do we really want to take them seriously enough to require that they shall be educated and trained for so many years in what corresponds to our technical schooling? How far do such practitioners of a substitute for medicine really compete with us? If competition must be considered, will it be increased or diminished by educating charlatans? If charlatans make work for the regular profession, will the public be safe-guarded by requiring that the various psychic and physical means employed be drilled in by longer terms of study, and that the irregular practitioners use better grammar, know more history, etc? Certainly something should be done and we would be glad to print the views of our readers.

